# Exploring Risk Perception, Mental Health, Mental Fatigue, Stigma, and the Quality of Life among UAE Healthcare Workers during the COVID-19 Pandemic: A National Multicentric Cross-Sectional Study

**DOI:** 10.3390/ijerph21091124

**Published:** 2024-08-26

**Authors:** Yousef M. Aljawarneh, Nariman Ghader, Ahmad M. Al-Bashaireh, Heyam F. Dalky, Hasan Al-Omari, Osama Alkouri, Sarah R. Sanad, Noor Al Mheiri, Aji Gopakumar, Sara AlShaya, Gregory L. Blatch, Hana Y. Ghunaim

**Affiliations:** 1 School of Nursing, Faculty of Health Sciences, Higher Colleges of Technology, Abu Dhabi P.O. Box 25026, United Arab Emirates; aalbashaireh@hct.ac.ae (A.M.A.-B.);; 2 Department of Mental Health, Medical Services Sector, Emirates Health Services, Dubai P.O. Box 2299, United Arab Emirates; nariman.ghader@ehs.gov.ae (N.G.); noor.majed@ehs.gov.ae (N.A.M.); 3 Department of Community and Mental Health Nursing, Faculty of Nursing, WHO Collaborating Center, Jordan University of Science & Technology, Irbid 22110, Jordan; hfdalky@just.edu.jo; 4 Department of Community and Mental Health Nursing, Faculty of Nursing, The Hashemite University, Zarqa P.O. Box 13115, Jordan; h.alomari@hu.edu.jo; 5 Faculty of Nursing, Yarmouk University, Irbid P.O. Box 566, Jordan; o.alkouri@yu.edu.jo; 6 Data and Statistics Department (DSD), Emirates Health Services (EHS), Dubai P.O. Box 2299, United Arab Emirates; aji.gopakumar@ehs.gov.ae (A.G.); sara.alshaya@ehs.gov.ae (S.A.); 7 The Vice Chancellery, The University of Notre Dame Australia, P.O. Box 1225, Fremantle, WA 6959, Australia; greg.blatch@nd.edu.au; 8 Fatima College of Health Sciences, Institute of Applied Technology, Al Ain P.O. Box 24162, United Arab Emirates; hana.ghunaim@actvet.gov.ae

**Keywords:** COVID-19, healthcare workers, mental fatigue, mental health, psychological impact, quality of life, risk perception, stigma, United Arab Emirates

## Abstract

Globally, the COVID-19 pandemic has presented serious mental health challenges for healthcare professionals. This study investigated the mental health, mental fatigue, quality of life, and stigma of social discrimination among healthcare workers in the United Arab Emirates (UAE) during the COVID-19 pandemic. A correlational, cross-sectional, multi-centric design was employed to collect data from 1383 healthcare workers across various healthcare settings. Participants were recruited using combined cluster and purposive sampling techniques. Standardized questionnaires, including the COVID-19 Pandemic Mental Health Questionnaire (CoPaQ), the Mental Fatigue Scale (MFS), the Social Discrimination Scale-Stigma Subscale (SDS), and the WHO Quality of Life Questionnaire-Brief (WHOQOL-BREF), were administered to assess the study variables. The results indicated significant mental health impacts, with high average scores for post-traumatic stress disorder (PTSD) (9.37 ± 6.74) and positive coping by inner strengths (17.63 ± 5.72). Mental fatigue was prevalent (8.15 ± 8.62), and stigma of social discrimination scored notably (23.83 ± 7.46). Quality of life was the highest in the social domain (65.38 ± 24.58). Significant correlations were observed between mental health subscales, mental fatigue, and quality of life domains. These findings highlight the critical need for targeted mental health support programs, improved social support networks, and personalized interventions to mitigate the mental health challenges faced by healthcare workers. Healthcare organizations can guarantee a resilient workforce that can handle future health crises by giving mental health resources and support systems top priority.

## 1. Introduction

The Coronavirus disease 2019 (COVID-19) was declared a pandemic and a major public health crisis globally in March 2020 [1,2]. As of early August 2022, 581.8 million confirmed cases and 6.4 million deaths have been reported globally [3]. Healthcare workers were among the most vulnerable groups during the pandemic, facing occupational hazards, fear of transmitting the virus to family members, longer shifts, increased cases and death rates, lack of medical equipment, and isolation restrictions [3,4,5,6,7,8,9]. These factors contributed to mental health issues such as fear, anxiety, stress, anger, and depression [10,11,12,13,14,15,16,17]. A recent study found that 61% of physicians experienced burnout, 57% reported anxiety, tearfulness, or anger, 46% experienced social isolation, and over 55% knew of a doctor who had attempted or committed suicide [18].

Mental health disorders lead to a poor quality of life (QoL), mental fatigue, and stigmatization against those infected [7,19]. QoL is a multidimensional concept reflecting people’s perception of their culture and is influenced by various factors, including health status [20,21,22]. Mental fatigue, characterized by lack of motivation and tiredness, affects cognitive performance, concentration, and planning, and is a symptom of psychiatric disorders like anxiety and depression [23,24,25]. Health professionals experienced greater levels of mental fatigue, anxiety, and depression during COVID-19 [26,27].

Stigmatization, related to caring for patients with contagious diseases, contributed to uncertainty, stereotypes, and discrimination [28]. Social stigma in healthcare refers to the negative association with individuals who have a particular disease, leading to labeling and discrimination against those affected by COVID-19 [29,30]. This stigma can result in healthcare workers fearing virus contraction and feeling guilty about potentially spreading it to their families, ultimately affecting the quality of care provided [28,31,32]. Stigmatization leads to reluctance in seeking healthcare, hampered pandemic control, and increased stress, anxiety, and burnout among healthcare professionals [33,34,35].

As of 14 October 2023, the United Arab Emirates (UAE) reported 1,067,030 diagnosed COVID-19 cases with nearly 2349 deaths [36]. Despite the existing research on the mental and psychological impacts of COVID-19 in the UAE, there remains a significant gap in understanding the comprehensive effects on mental health, mental fatigue, social stigma, and quality of life (QoL) among healthcare workers during the pandemic. Our study aimed to address this gap by providing a detailed analysis of these factors within the unique healthcare environment of the UAE.

## 2. Materials and Methods

### 2.1. Enrolment and Data Collection

This national correlational, cross-sectional study was conducted in the United Arab Emirates to evaluate the risk perception, mental health, mental fatigue, quality of life, and stigma among healthcare frontliners during the COVID-19 pandemic. Participants were recruited using cluster and purposive sampling techniques from various healthcare settings, including those under the Ministry of Health and Prevention (MOHAP), Abu-Dhabi Health Services Company (SEHA), Sheikh Shakhbout Medical City (SSMC), and the private sector. The study received ethical approval from multiple research ethics committees: Higher Colleges of Technology (HCT) (REIC-06-2021), Ministry of Health and Prevention (MOHAP/DXB-REC/M.M.M/No.33/2022), SEHA Research Ethics Committee-SEHA Research Oversight Committee (HREC SEHA-IRB-42), and SSMC (SSMCREC-310). Data were collected in two phases between 18 March and 10 June 2022.

### 2.2. Pilot Phase

The pilot phase (18–24 March) tested the reliability and validity of the study instruments on 50 healthcare workers. The COVID-19 Pandemic Mental Health Questionnaire (CoPaQ), Mental Fatigue Scale (MFS), Social Discrimination Scale (SDS), and WHO Quality of Life Questionnaire-Brief (WHOQOL-BREF) were used, along with a Demographic Data Questionnaire. A priori criterion of Cronbach’s alpha ≥ 0.70 was used for adequate evidence of each scale and subscales reliability [37]. The pilot confirmed satisfactory internal consistency reliability (Cronbach’s alpha ≥ 0.70).

### 2.3. Data Collection

The main data collection phase began on 31 March and ended on 10 June 2022. Healthcare frontliners from the approved settings were invited to participate via institutional email. Inclusion and exclusion criteria were established to ensure a representative sample of healthcare workers for this study. Inclusion criteria were: (1) healthcare workers currently employed in the UAE, (2) adults aged 18 and above, (3) individuals who provided direct patient care during the COVID-19 pandemic, and (4) those who consented to participate in the study. Participation was voluntary, anonymous, and confidential. Out of 1443 invitations, 1383 participants completed the survey, forming the final sample.

### 2.4. Definitions of Key Terms

1. Risk Perception: Risk perception refers to an individual’s assessment of the likelihood and severity of a risk. In this study, it pertains to healthcare workers’ perceptions of their risk of contracting COVID-19 and the associated consequences.
2. Mental Health: Mental health encompasses emotional, psychological, and social well-being. It influences how individuals think, feel, and act, particularly under stress. In this study, mental health was assessed through symptoms of post-traumatic stress disorder (PTSD), anxiety, and general stress.
3. Mental Fatigue: Mental fatigue refers to the decline in cognitive performance and the feeling of exhaustion resulting from prolonged cognitive activity or stress. In this study, it was measured by assessing healthcare workers’ levels of cognitive and emotional exhaustion.
4. Stigma: Stigma refers to the negative attitudes and beliefs that lead to discrimination against individuals or groups. In this study, it pertains to the social discrimination experienced by healthcare workers due to their association with treating COVID-19 patients.
5. Quality of Life: Quality of life is a broad multidimensional concept that includes subjective evaluations of both positive and negative aspects of life. In this study, it was assessed in four domains: physical health, psychological state, social relationships, and environmental context.

### 2.5. Measurements

1. COVID-19 Pandemic Mental Health Questionnaire (CoPaQ): Participants’ risk perception, mental health impact, and positive coping were assessed using the COVID-19 Pandemic Mental Health Questionnaire (CoPaQ) [38]. The CoPaQ is a newly developed and highly comprehensive self-report measure of personal and social consequences of the COVID-19-pandemic with an application scope world-wide. The CoPaQ includes six sections with general questions as follows: general questions on the personal and social consequences of the COVID-19-pandemic (4 questions), general questions on the risk factors for a severe course of COVID-19 applied to participants (9 questions), general questions on the risk factors for a severe course of COVID-19 applied to participants the people living with participants in the same household (9 questions), 2 questions on the quarantine and work from home (WFH), nominal questions on previous diagnosis by a doctor or therapist with one or more of the psychological disorders (10 questions), and 1 question current receiving of psychotherapy. For the measurement of risk perception, mental health impact, and positive coping, 10 subscales were used as follows: COVID-19 contamination anxiety (9 questions), COVID-19 adherence measures (3 questions), COVID-19 mental health impact related to Post-Traumatic Stress Disorders (3 questions), COVID-19 mental health impact related to sleep disturbances (6 questions), COVID-19 mental health impact related to substance use (6 questions), COVID-19 mental health impact related to stress burdens (16 questions), COVID-19 positive coping by daytime structure (3 questions), COVID-19 positive coping by social contacts (4 questions), COVID-19 positive coping by inner strengths (7 questions), and COVID-19 stressor impact related to conspiracy beliefs (6 questions). Each subscale was measured on a Likert’s scale from 0 (not at all) to 4 (very much). Two scoring protocols were used for mental health impact subscales the continuous and the categorical scoring. For the continuous scoring, a sum score of all items pertaining to each subscale was calculated as an index of COVID-19-specific impact with higher scores indicating a greater stressor impact. For categorical scoring, using the total sum of all items, each subscale was categorized into 4 categories as follows: No Stressor Impact, Mild Stressor Impact, Moderate Stressor Impact, and Severe Stressor Impact. For mental health impact subscales, the continuous scoring protocol was used with higher scores indicating greater ability to cope with the COVID-19 stressors impact. In this study, the questionnaire demonstrated excellent internal consistency reliability with Cronbach’s alpha of 0.962.
2. Mental Fatigue Scale (MFS-14): Measured mental fatigue and related symptoms using 14 questions covering topics such as generalized fatigue, concentration, memory, slowness of thinking, stress sensitivity, and sleep problems. Each item had four response options: no (0), slight (1), fairly serious (2), and serious (3) problems. Scores above 10 indicated a problem with mental fatigue [39]. The MFS showed excellent internal consistency reliability (Cronbach’s alpha = 0.948).
3. Social Discrimination Scale-Stigma Subscale (SDS-12): Assessed stigma related to COVID-19 using 12 statements measuring personal and public perceptions of stigma. Participants rated each statement on a Likert scale from 1 (strongly disagree) to 4 (strongly agree). Higher scores indicated higher levels of stigma [40]. The SDS demonstrated excellent internal consistency reliability (Cronbach’s alpha = 0.901).
4. WHO Quality of Life Questionnaire (WHOQOL-BREF): Measured quality of life across four domains: Physical health, Psychological, Social relationships, and Environment. The questionnaire included 26 questions rated on a 1–5 Likert scale. Domain scores were scaled to a 0–100 scale for comparability [41]. The WHOQOL-BREF showed excellent internal consistency reliability (Cronbach’s alpha = 0.947).
5. Demographic Data Questionnaire: Collected social and demographic data from participants, including age, gender, nationality, educational level, occupational role, marital status, number of children, previous diagnosis of COVID-19, self-isolation, living conditions, and household status.

### 2.6. Data Analysis

Data were analyzed using SPSS (IBM SPSS Statistics, Version 29, 2022). Descriptive statistics were computed for continuous and categorical variables. The data was examined for statistical assumptions prior to each analysis. Independent samples *t*-tests and a one-way analysis of variance (ANOVA) were used to compare means across demographic and COVID-19-related variables. Pearson’s correlation coefficients assessed relationships between key study variables. Statistical significance was set at *p* < 0.05.

## 3. Results

### 3.1. Participants’ Demographics and COVID-19-Related Characteristics

Table 1 shows demographics and COVID-19-related characteristics. A total of 1383 healthcare workers were enrolled in the study. The participants’ mean age was 39.87 (SD = 8.76). The majority were females (75.1%), married (82%), non-Emirati (89.9%), had a diploma/bachelor’s degree (75.1%), employed (98.93%), nurses (75.6%), had more than ten years of experience (54.7%), with income less or equal to 10,000 Dirhams (60%), and either living or working in city of Sharjah (46%, 52.2%, respectively). Concerning COVID-19, 61.6% reported an ever-positive PCR test; however, only 12.4% reported COVID-19 symptoms, 5% were under quarantine, and 12.7% were working/studying remotely from their home.

### 3.2. Mental Health, Mental Fatigue, Stigma of Social Discrimination, and Quality of Life

Participants’ total scores of mental health impacts, mental fatigue, the stigma of social discrimination, and quality of life were reported in Table 2. Concerning mental health impacts for risk perception, while considering the number of response items, the highest average total score was COVID-19 mental health impact related to contamination anxiety (12.57 ± 9.14), followed by 19 mental health impact related to PTSD (9.37 ± 6.74). Furthermore, for COVID-19 positive coping, the highest average score was for COVID-19 positive coping by inner strengths (17.63 ± 5.72). The average total score of mental fatigue and stigma of social discrimination were 8.15 ± 8.62 and 23.83 ± 7.46, respectively. The highest reported quality of life score was for the social domain (65.38 ± 24.58), followed by the psychological domain (62.69 ± 20.30).

### 3.3. Levels of COVID-19 Contamination Anxiety, Stress Burden, and Mental Health Impacts

Table 3 presents the distribution of stressor impacts among healthcare workers for several key variables: COVID-19 contamination anxiety, COVID-19 mental health impacts (including PTSD, sleep disturbances, and substance use), and COVID-19 stress burden. Regarding COVID-19 contamination anxiety, the majority of participants reported no stressor impact (43.5%), while 31.4% experienced mild stressor impact. Moderate and severe stressor impacts were reported by 18.0% and 7.2% of participants, respectively. This distribution highlights that while a substantial portion of the healthcare workers experienced some level of anxiety related to contamination, a significant number reported only mild to no stressor impact. Similarly, most participants indicated no stressor impact related to PTSD (43.8%). Mild PTSD symptoms were reported by 34.9% of participants, with moderate and severe impacts observed in 16.0% and 5.3%, respectively. Regarding COVID-19 mental health impact related to sleep disturbances, the most frequent category was no stressor impact (64.3%), with mild disturbances reported by 18.9% of participants. Moderate and severe sleep disturbances were less common, affecting 12.9% and 3.9% of participants, respectively. These findings indicate that sleep disturbances were less prevalent compared to other mental health impacts. In contrast, a significant majority reported no stressor impact related to substance use (71.8%). Mild impacts were observed in 18.2% of participants, while moderate and severe impacts were reported by 8.5% and 1.4%, respectively. This indicates that substance use was relatively less of a concern compared to other stressors. Regarding COVID-19 stress burden, the distribution shows the varying levels of stress experienced by healthcare workers, with a notable portion reporting mild to moderate stress burdens.

### 3.4. Inferential Analysis of Tested Variables on Participants’ Sociodemographic Variables

#### 3.4.1. Independent Sample *T*-Test to Compare the Means of Tested Variables across Participants’ Sociodemographic Variables

Table 4 shows the means of total scores of scales and subscales of mental health impacts, mental fatigue, stigma of social discrimination, and quality of life across the participants’ sociodemographic. Compared with females, males reported a lower level of COVID-19 adherence measures (8.06 ± 2.83 vs. 8.50 ± 2.61, *p* = 0.009); however, males reported a higher level of COVID-19 stressor impact related to conspiracy beliefs (10.93 ± 5.45 vs. 10.20 ± 5.61, *p* = 0.037), physical (64.62 ± 21.84 vs. 59.82 ± 21.65, *p* ˂ 0.001), psychological (65.36 ± 20.52 vs. 61.80 ± 20.15, *p* = 0.005), social (68.07 ± 23.88 vs. 64.49 ± 24.75, *p* = 0.019), and environmental (65.67 ± 19.41 vs. 61.65 ± 19.98, *p* = 0.001) quality of life. The rest of the comparison for the significant differences in scales and subscales across the demographics and COVID-19 related characteristics were reported in Table 4. Furthermore, non-significant differences in outcome variables across the demographics were not reported in Table 4.

#### 3.4.2. One-Way ANOVA to Compare the Means of Tested Variables across Participants’ Sociodemographic Characteristics

The one-way ANOVA test was performed to compare the differences in means of the total scores of tested variables across participants’ demographics, profession, city of residency, and city of employment (Table 5). Regarding COVID-19 adherence measures, significant differences were observed (F = 9.703, *p* < 0.001) among physicians, nurses, and others, indicating that adherence to COVID-19 measures varied by profession, with nurses showing different adherence patterns compared to physicians and other professionals. Furthermore, the results showed significant differences in impact of PTSD related to COVID-19 across the participants profession (F = 11.718, *p* < 0.001), with nurses experiencing more significant impacts compared to physicians and other professions. Similarly, a significant variation in stress burden related to COVID-19 (F = 8.004, *p* < 0.001) was found across professions, with nurses reporting higher stress levels. Regarding Quality-of-Life variables, significant differences were found in physical (F = 8.553, *p* < 0.001), psychological (F = 11.871, *p* < 0.001), social (F = 11.055, *p* < 0.001), and environmental (F = 21.742, *p* < 0.001) domains across the professions, suggesting that professionals experienced varied quality of life impacts based on their role, with significant differences in perceived quality across the various domains. On the other hand, significant differences in COVID-19 mental health impact related to PTSD (F = 3.034, *p* = 0.004), COVID-19 stress burden (F = 3.356, *p* = 0.001), COVID-19 positive coping (F = 2.398, *p* = 0.019), stigma of social discrimination (F = 3.036, *p* = 0.004) and QoL variables were found based on city of residency, indicating varied impacts among residents of different cities, with residents of certain cities reporting higher impacts. Similarly, the differences were noted in the tested variables based on the city of employment. For instance, significant differences in COVID-19 mental health impact related to PTSD (F = 2.266, *p* = 0.027) highlight that the impact of PTSD varied based on the city of employment, with some cities showing higher impacts. Furthermore, non-significant differences in outcome variables across the demographics were not reported in Table 5.

### 3.5. Pearsons’ Correlation Analysis for the Relationships between the Outcome Variables

Table 6 presents the bivariate relationships between COVID-19 mental health impacts, mental fatigue, stigma of social discrimination, and quality of life. Also, age and number of internet hours were included in this table. Age exhibited several statistically significant correlations. Specifically, it negatively correlated with contamination anxiety, sleep disturbances, substance use, stress burden, and mental fatigue, suggesting that older participants reported lower levels of these stressors. Conversely, age was positively correlated with daytime structure and social contact, indicating that older individuals might have better-organized daily routines and more social interactions. Furthermore, contamination anxiety showed a strong positive correlation with PTSD (r = 0.544, *p* < 0.01), sleep disturbances (r = 0.551, *p* < 0.01), and substance use (r = 0.543, *p* < 0.01), highlighting the significant impact of contamination anxiety on various mental health aspects. Contamination anxiety also had notable positive correlations with stress burden (r = 0.633, *p* < 0.01) and stigma of social discrimination (SSD) (r = 0.180, *p* < 0.01). Additionally, adherence to measures was positively correlated with contamination anxiety (r = 0.222, *p* < 0.01) and had moderate correlations with PTSD (r = 0.277, *p* < 0.01) and stress burden (r = 0.222, *p* < 0.01). This suggests that adherence measures are associated with higher levels of anxiety and stress but show weaker relationships with other variables. PTSD was strongly correlated with sleep disturbances (r = 0.686, *p* < 0.01), and substance use (r = 0.686, *p* < 0.01). The strong associations indicate that PTSD symptoms are prevalent among those experiencing high levels of anxiety, sleep issues, and substance use. Sleep disturbances was positively correlated with PTSD (r = 0.693, *p* < 0.01), substance use (r = 0.837, *p* < 0.01), and stress burden (r = 0.680, *p* < 0.01), suggesting that individuals with sleep disturbances experience significant stress and increased substance use. Stress burden showed strong correlations with PTSD (r = 0.681, *p* < 0.01), sleep disturbances (r = 0.680, *p* < 0.01), and substance use (r = 0.739, *p* < 0.01), indicating a robust relationship between perceived stress and other mental health issues. Regarding Quality of Life (QoL) variables, All QoL subscales (physical, psychological, social, environmental) showed negative correlations with mental health stressors such as contamination anxiety, PTSD, and stress burden. For instance, QoL Physical (QOL-PH) was negatively correlated with contamination anxiety (r = −0.326, *p* < 0.01), PTSD (r = −0.302, *p* < 0.01), and stress burden (r = −0.422, *p* < 0.01), indicating that higher levels of anxiety and stress are associated with poorer quality of life. Mental fatigue exhibited significant positive correlations with contamination anxiety (r = 0.434, *p* < 0.01), PTSD (r = 0.381, *p* < 0.01), sleep disturbances (r = 0.545, *p* < 0.01), and substance use (r = 0.491, *p* < 0.01). These correlations suggest that increased levels of mental fatigue are strongly associated with higher levels of anxiety, PTSD symptoms, sleep issues, and substance use. Additionally, mental fatigue showed moderate positive correlations with stress burden (r = 0.479, *p* < 0.01) and a weaker but still significant correlation with stigma of social discrimination (r = 0.186, *p* < 0.01), indicating that mental fatigue is linked to perceived stress and stigma but to a lesser extent. Regarding stigma of social discrimination (SSD), there was a weak correlation with mental fatigue (r = 0.186, *p* < 0.01) and was negatively correlated with several QoL variables, including physical (r = −0.355, *p* < 0.01) and psychological (r = −0.424, *p* < 0.01), indicating that higher stigma is associated with a lower quality of life across different domains.

## 4. Discussion

This study aimed to investigate the mental health, mental fatigue, quality of life and stigma of social discrimination among healthcare workers during the COVID-19 pandemic in United Arab Emirates. The study enrolled a total of 1383 healthcare workers of them, 61.6% had tested positive for the virus at some point during the outbreak of the pandemic. The results of our study revealed noteworthy effects on the mental health of healthcare professionals. Among these effects, the COVID-19 mental health impact associated with PTSD had the highest average score. This finding is consistent with earlier research reporting that healthcare workers experienced higher levels of PTSD symptoms during the pandemic [42,43]. Local studies in the UAE have also reported similar trends. For instance, Al Dhaheri et al. (2020) found high levels of psychological distress, including PTSD, among healthcare professionals in Abu Dhabi [44]. Additionally, a study by Cheikh Ismail et al. (2021) highlighted significant levels of anxiety and depression among healthcare workers in the UAE during the pandemic, reinforcing our findings on mental health impacts [45]. Furthermore, the high average score for positive coping by inner strengths indicates a resilient response from the participants, which has also been observed in studies highlighting the crucial role of positive coping mechanisms [13,46]. The study results show that healthcare workers face significant challenges related to mental fatigue and social discrimination. These findings align with other studies that emphasize the dual burden healthcare workers endure [46,47]. For example, a study by Saddik et al. (2021) on healthcare workers in Dubai reported significant levels of mental fatigue and social discrimination, similar to our findings [48]. According to quality-of-life scores in this study, the psychological domain scored lower than the social domain. This is consistent with other research demonstrating the critical role social and psychological support networks play in preserving quality of life when facing major stressors [49,50].

A significant proportion of participants in our study reported experiencing moderate to severe impacts from various COVID-19-related stressors, notably contamination anxiety, PTSD, and general stress burdens. These results are consistent with earlier studies that showed the pandemic had a significant psychological toll on healthcare workers [13,51,52]. Interestingly, our study found that relatively fewer participants reported moderate to severe impacts from sleep disturbances and substance use. This indicates that while some aspects of mental health are significantly affected by the pandemic, others may show greater variation [53].

In our study, the independent *t*-tests and one-way ANOVA analyses showed notable variations in the effects of mental health and quality of life among different demographic groups. For example, men reported a better quality of life in all domains and lower levels of COVID-19 adherence measures, but higher levels of stressor impacts related to conspiracy beliefs. Specifically, men had higher scores in physical, psychological, social, and environmental domains compared to women, who had lower scores in all these domains. However, men reported higher stressor impacts related to conspiracy beliefs, with an average score of compared to in women. These gender differences are consistent with earlier research showing that men and women react to pandemic-related stressors differently [54,55]. Furthermore, our study revealed notable variations among occupations and places of employment, indicating that the professional role and workplace atmosphere have a substantial impact on mental health and overall well-being [55]. Specifically, nurses reported higher levels of mental fatigue and social discrimination compared to physicians, who had lower scores in these areas. Similarly, healthcare workers in high-risk areas such as emergency departments and COVID-19 wards reported higher PTSD scores compared to those in low-risk areas. Therefore, understanding these variations is pivotal to putting in place support networks and interventions that are specifically designed to meet the needs of every healthcare subgroup.

The correlation analysis found multiple significant correlations shedding light on the interconnected nature of mental health issues and quality of life domains among healthcare workers. Notably, the strong correlation between mental health impacts related to sleep disturbances and substance use is consistent with earlier study indicating that individuals experiencing sleep disturbances may be more susceptible to substance use as a coping mechanism [56,57,58]. Additionally, we found a strong correlation between the environmental and physical quality of life domains, demonstrating how these domains are interrelated in determining overall well-being [59,60].

As we outlined above, while many findings in our study align with existing literature, several unique insights emerged that contribute to the scientific understanding of healthcare workers’ experiences during the pandemic. For instance, our study found that nurses, especially those from high-risk regions were reported to have higher levels of mental fatigue and social discrimination compared to physicians. This clearly indicates that there is an urgent need for specialized mental health treatments for nurses working under pressure. Furthermore, our research identified gender-based stressor effects whereby males had greater levels of tension than their female counterparts owing to conspiracy belief-related issues. This finding about behavioral variations between the genders regarding stress responses indicates how crucial it is when developing programs aimed at improving mental wellbeing across genders.

The study’s strengths include its comprehensive evaluation of multiple mental health and quality of life domains in addition to its large sample size. The findings’ generalizability is further improved by the participation of a diverse range of healthcare professionals from various cities and units. Moreover, the data reported in this study could be a good comparable source for other researchers and future studies that aim to study such a large number of variables simultaneously. However, the study does have certain limitations. Inferring causality between variables is limited by the cross-sectional design. Due to participant under- or overreporting of symptoms and experiences, self-reported data may introduce bias. Furthermore, there may be limited generalizability to other regions due to the study’s execution within a particular cultural and geographical context.

The findings of our study have significant implications for post-COVID situations and future healthcare crises. First, the high levels of mental fatigue and social discrimination reported by healthcare workers, particularly nurses in high-risk areas, suggest that ongoing mental health support and interventions will be crucial even after the immediate threat of the pandemic has subsided. Establishing long-term mental health programs that address these specific stressors can help mitigate the prolonged impact on healthcare workers’ well-being. Moreover, the gender-specific differences in stressor impacts indicate that mental health interventions should be tailored to address the unique needs of different demographic groups. Our study also highlights the importance of strengthening social support networks within healthcare settings. Enhanced peer support programs, resilience training, and opportunities for professional development can help create a more supportive work environment that can buffer against the negative impacts of high-stress situations.

The results of the study recommend that specific mental health support programs for healthcare workers that address stress management, PTSD, and fatigue reduction are imperative. In healthcare settings, social support networks should be strengthened in order to reduce feelings of isolation and enhance mental health. Positive coping strategies like mindfulness and resilience training can also be promoted. Furthermore, customized interventions that cater to the particular requirements of various professional and demographic groups are advised. For instance, targeted support for nurses in high-risk areas who experience higher levels of mental fatigue and social discrimination, and tailored programs for men who report higher stressor impacts related to conspiracy beliefs, could be beneficial. In order to maintain a robust and efficient healthcare workforce that is able to handle both present and future health crises, policymakers and administrators must prioritize mental health resources and support networks. These actions have a substantial impact on healthcare policy and practice.

## 5. Conclusions

This national study sheds light on the significant mental health challenges faced by healthcare workers during the COVID-19 pandemic, focusing on mental health, mental fatigue, quality of life and social discrimination. Despite showing resilience through positive coping strategies, healthcare workers are still prone to experiencing high levels of stress, anxiety, and other mental health and mental fatigue issues. The study stresses the importance of addressing these challenges through targeted support programs, improved social support networks, and personalized interventions tailored to the unique needs of different groups within the healthcare workforce. By prioritizing mental health resources and support systems, healthcare organizations can better care for their employees, ensuring their well-being and ability to effectively handle current and future health emergencies.

## Figures and Tables

**Table 1:** Participant demographics and COVID-19-related characteristics.

Variable	n (%) or Mean ± SD
Age	39.87 ± 8.76
Gender	
Male	344 (24.9)
Female	1039 (75.1)
Marital Status	
Married	1134 (82.0)
Unmarried	249 (18.0)
Nationality	
Emirati	140 (10.1)
Non-Emirati	1243 (89.9)
Education Level	
Diploma and Bachelor	1038 (75.1)
Master and PhD	345 (24.9)
Employment Status	
Employed	1360 (98.3)
Not employed	23 (1.7)
Profession	
Physician	229 (16.6)
Nurse	1045 (75.6)
Laboratory Specialist/Technician	10 (0.7)
Imaging Specialist/Technician	5 (0.4)
Midwife	4 (0.3)
Social Worker	5 (0.4)
Pharmacist	17 (1.2)
Other Allied HCP	60 (4.3)
Years of Experience	
≤10 years	627 (45.3)
>10 years	756 (54.7)
Income (AED)	
≤10,000 Dirhams	830 (60.0)
>10,000 Dirhams	553 (40.0)
City of Residency	
Dubai	174 (12.6)
Abu Dhabi	207 (15.0)
Sharjah	636 (46.0)
Fujairah	109 (7.9)
Umm Al Quwain	45 (3.3)
Ras Al Khaimah	57 (4.1)
Ajman	105 (7.6)
Al-Ain	50 (3.6)
City of Employment	
Dubai	176 (12.7)
Abu Dhabi	211 (15.3)
Sharjah	722 (52.2)
Fujairah	96 (6.9)
Umm Al Quwain	45 (3.3)
Ras Al Khaimah	52 (3.8)
Ajman	33 (2.4)
Al-Ain	48 (3.5)
Suffer from COVID-19 Symptoms	
Yes	172 (12.4)
No	1200 (86.8)
Don’t Know	11 (0.8)
Ever PCR Positive for COVID-19	
Yes	852 (61.6)
No	528 (38.2)
Don’t Know	3 (0.2)
Under Quarantine	
Yes	69 (5.0)
No	1314 (95.0)
Working/studying Remotely from Home	
Yes	176 (12.7)
No	1207 (87.3)

Note: AED = Arab Emirates Dirham, COVID-19 = corona virus disease, HCP = healthcare workers, n = sample, SD = standard deviation.

**Table 2:** Descriptive statistics for the continuous scoring of mental health, mental fatigue, stigma of social discrimination, and quality of life.

Scale/Subscale	Mean ± SD
COVID-19 Mental Health Questionnaire (CoPaQ)	
COVID-19 Contamination Anxiety	12.57 ± 9.14
COVID-19 Adherence Measures	8.39 ± 2.67
COVID-19 Mental Health Impact: PTSD	9.37 ± 6.74
COVID-19 Mental Health Impact: Sleep Disturbances	5.56 ± 6.46
COVID-19 Mental Health Impact: Substance Use	4.42 ± 5.52
COVID-19 Stress Burden	18.21 ± 15.10
COVID-19 Positive Coping: Daytime Structure	5.75 ± 2.60
COVID-19 Positive Coping: Social Contacts	7.71 ± 3.74
COVID-19 Positive Coping: Inner Strength	17.63 ± 5.72
COVID-19: Internet Hours	1.86 ± 2.62
COVID-19: Conspiracy Beliefs	10.38 ± 5.57
Mental Fatigue	8.15 ± 8.62
Stigma of Social Discrimination	23.83 ± 7.46
Quality of Life (QOL)	
Physical Domain	61.02 ± 21.79
Psychological Domain	62.69 ± 20.30
Social Domain	65.38 ± 24.58
Environmental Domain	62.65 ± 19.91

Note: COVID-19 = corona virus disease, PTSD = post-traumatic stress disorder, SD = standard deviation.

**Table 3:** Descriptive statistics for categorical scoring of mental health, mental fatigue, stigma of social discrimination, and quality of life.

Variable		n (%)
COVID-19 Contamination Anxiety	No Stressor Impact	601 (43.5)
	Mild Stressor Impact	434 (31.4)
	Moderate Stressor Impact	249 (18.0)
	Severe Stressor Impact	99 (7.2)
COVID-19 Mental Health Impact: PTSD	No Stressor Impact	606 (43.8)
	Mild Stressor Impact	483 (34.9)
	Moderate Stressor Impact	221 (16.0)
	Severe Stressor Impact	73 (5.3)
COVID-19 Mental Health Impact: Sleep Disturbances	No Stressor Impact	889 (64.3)
	Mild Stressor Impact	261 (18.9)
	Moderate Stressor Impact	179 (12.9)
	Severe Stressor Impact	54 (3.9)
COVID-19 Mental Health Impact: Substance Use	No Stressor Impact	993 (71.8)
	Mild Stressor Impact	252 (18.2)
	Moderate Stressor Impact	118 (8.5)
	Severe Stressor Impact	20 (1.4)
COVID-19 Stress Burden	No Stressor Impact	718 (51.9)
	Mild Stressor Impact	388 (28.1)
	Moderate Stressor Impact	215 (15.5)
	Severe Stressor Impact	62 (4.5)

Note: COVID-19 = corona virus disease, n = sample, PTSD = post-traumatic stress disorder.

**Table 4:** Independent sample *t*-test of the tested variables on participants’ sociodemographic variables.

Outcome Variable	Sociodemographic Variable	Mean ± SD	df	*T*-Value	*p*-Value
	Gender (n: 344 male, 1039 female)		1381		
COVID-19 Adherence Measures	Male	8.06 ± 2.83		−2.620	0.009 **
	Female	8.50 ± 2.61			
COVID-19: Internet Hours	Male	2.22 ± 2.89		2.741	0.006 **
	Female	1.74 ± 2.51			
COVID-19: Conspiracy Beliefs	Male	10.93 ± 5.45		2.091	0.037 *
	Female	10.20 ± 5.61			
QOL: Physical	Male	64.62 ± 21.84		3.557	<0.001 ***
	Female	59.82 ± 21.65			
QOL: Psychological	Male	65.36 ± 20.52		2.826	0.005 **
	Female	61.80 ± 20.15			
QOL: Social	Male	68.07 ± 23.88		2.350	0.019 *
	Female	64.49 ± 24.75			
QOL: Environmental	Male	65.67 ± 19.41		3.254	0.001 ***
	Female	61.65 ± 19.98			
	Marital status (n: 1134 married, 249 unmarried)		1381		
COVID-19 Contamination Anxiety	Married	12.17 ± 8.98		−3.452	0.001 ***
	Unmarried	14.37 ± 9.64			
COVID-19 Mental Health Impact: Sleep Disturbances	Married	5.09 ± 6.23		−5.391	<0.001 ***
	Unmarried	7.71 ± 7.07			
COVID-19 Mental Health Impact: Substance Use	Married	4.13 ± 5.37		−3.981	<0.001 ***
	Unmarried	5.77 ± 5.99			
COVID-19 Positive Coping: Daytime Structure	Married	5.86 ± 2.55		3.960	0.001 ***
	Unmarried	5.26 ± 2.77			
Mental Fatigue	Married	7.60 ± 8.36		−5.056	<0.001 ***
	Unmarried	10.63 ± 9.33			
QOL: Physical	Married	62.15 ± 21.59		4.115	<0.001 ***
	Unmarried	55.84 ± 21.98			
QOL: Psychological	Married	63.57 ± 20.05		3.443	0.001 ***
	Unmarried	58.69 ± 20.96			
QOL: Social	Married	66.59 ± 24.33		3.947	<0.001 ***
	Unmarried	59.84 ± 24.99			
QOL: Environmental	Married	63.14 ± 19.70		1.960	0.050 *
	Unmarried	60.42 ± 20.73			
	Nationality (n: 140 Emirati, 1243 Non-Emirati)		1381		
COVID-19 Contamination Anxiety	Emirati	15.34 ± 8.88		3.801	<0.001 ***
	Non-Emirati	12.25 ± 9.12			
COVID-19 Mental Health Impact: Sleep Disturbances	Emirati	8.11 ± 6.09		4.646	<0.001 ***
	Non-Emirati	5.28 ± 6.35			
COVID-19 Mental Health Impact: Substance Use	Emirati	6.35 ± 6.11		3.985	<0.001 ***
	Non-Emirati	4.20 ± 5.41			
COVID-19 Stress Burden	Emirati	21.42 ± 16.01		2.518	0.013 *
	Non-Emirati	17.85 ± 14.96			
COVID-19: Internet Hours	Emirati	1.36 ± 2.05		−2.429	0.003 **
	Non-Emirati	1.92 ± 2.67			
COVID-19: Conspiracy Beliefs	Emirati	11.46 ± 5.21		2.424	0.015 *
	Non-Emirati	10.26 ± 5.60			
Mental Fatigue	Emirati	10.85 ± 9.21		3.688	<0.001 ***
	Non-Emirati	7.84 ± 8.50			
Stigma of Social Discrimination	Emirati	26.95 ± 7.61		5.274	<0.001 ***
	Non-Emirati	23.47 ± 7.37			
QOL: Physical	Emirati	57.25 ± 20.64		−2.160	0.031 *
	Non-Emirati	61.44 ± 21.88			
	Education (n: 1038 Bachelor/Diploma, 345 Master/PhD)		1381		
COVID-19 Adherence Measures	Diploma and Bachelor	8.53 ± 2.61		3.401	0.001 ***
	Master and PhD	7.97 ± 2.82			
COVID-19 Mental Health Impact: PTSD	Diploma and Bachelor	9.84 ± 6.74		4.504	<0.001 ***
	Master and PhD	7.97 ± 6.53			
COVID-19 Mental Health Impact: Sleep Disturbances	Diploma and Bachelor	5.76 ± 6.54		1.992	0.047 *
	Master and PhD	4.98 ± 6.20			
COVID-19 Mental Health Impact: Substance Use	Diploma and Bachelor	4.60 ± 5.57		2.113	0.035 *
	Master and PhD	3.88 ± 5.35			
COVID-19 Stress Burden	Diploma and Bachelor	19.28 ± 15.18		4.576	<0.001 ***
	Master and PhD	15.01 ± 14.42			
COVID-19 Positive Coping: Inner Strength	Diploma and Bachelor	17.34 ± 5.90		−3.507	<0.001 ***
	Master and PhD	18.50 ± 5.06			
Stigma of Social Discrimination	Diploma and Bachelor	8.23 ± 8.61		2.753	0.006 **
	Master and PhD	7.91 ± 8.66			
QOL: Physical	Diploma and Bachelor	60.15 ± 21.78		−2.557	0.011 *
	Master and PhD	63.61 ± 21.63			
QOL: Psychological	Diploma andBachelor	61.49 ± 20.33		−3.821	<0.001 ***
	Master and PhD	66.29 ± 19.79			
QOL: Social	Diploma and Bachelor	64.19 ± 24.72		−3.136	<0.001 ***
	Master and PhD	68.96 ± 23.82			
QOL: Environmental	Diploma and Bachelor	60.93 ± 19.81		−5.632	<0.001 ***
	Master and PhD	67.83 ± 19.33			
	Income (n: 830 ≤ 10,000 Dirhams, 553 > 10,000 Dirhams)		1381		
COVID-19 Contamination Anxiety	≤10,000 Dirhams	15.34 ± 8.88		3.801	<0.001 ***
	>10,000 Dirhams	12.25 ± 9.12			
COVID-19 Mental Health Impact: PTSD	≤10,000 Dirhams	9.84 ± 6.74		4.504	<0.001 ***
	>10,000 Dirhams	7.97 ± 6.53			
COVID-19 Stress Burden	≤10,000 Dirhams	19.28 ± 15.18		4.576	<0.001 ***
	>10,000 Dirhams	15.01 ± 14.42			
COVID-19 Positive Coping: Inner Strength	≤10,000 Dirhams	17.34 ± 5.90		−3.507	<0.001 ***
	>10,000 Dirhams	18.50 ± 5.06			
QOL: Physical	≤10,000 Dirhams	60.15 ± 21.78		−2.557	0.011 *
	>10,000 Dirhams	63.61 ± 21.63			
QOL: Psychological	≤10,000 Dirhams	61.49 ± 20.33		−3.821	<0.001 ***
	>10,000 Dirhams	66.29 ± 19.79			
QOL: Social	≤10,000 Dirhams	64.19 ± 24.72		−3.136	<0.001 ***
	>10,000 Dirhams	68.96 ± 23.82			
QOL: Environmental	≤10,000 Dirhams	60.93 ± 19.81		−5.632	<0.001 ***
	>10,000 Dirhams	67.83 ± 19.33			
	Experience (n: 627 ≤ 10 years, 756 > 10 years)		1381		
COVID-19 Contamination Anxiety	≤10 years	13.19 ± 9.18		2.315	0.021 *
	>10 years	12.05 ± 9.08			
COVID-19 Mental Health Impact: Sleep Disturbances	≤10 years	6.11 ± 6.71		2.832	0.005 **
	>10 years	5.11 ± 6.22			
COVID-19 Mental Health Impact: Substance Use	≤10 years	4.95 ± 5.67		3.249	0.001 ***
	>10 years	3.98 ± 5.35			
COVID-19 Stress Burden	≤10 years	20.08 ± 15.39		4.225	<0.001 ***
	>10 years	16.66 ± 14.69			
COVID-19 Positive Coping: Daytime Structure	≤10 years	5.55 ± 2.72		−2.617	0.009 **
	>10 years	5.92 ± 2.48			
COVID-19 Positive Coping: Inner Strength	≤10 years	17.03 ± 6.07		−3.541	<0.001 ***
	>10 years	18.13 ± 5.38			
Mental Fatigue	≤10 years	8.08 ± 8.77		2.548	<0.011 *
	>10 years	7.61 ± 8.46			
Stigma of Social Discrimination	≤10 years	24.48 ± 7.65		2.970	0.003 **
	>10 years	23.28 ± 7.27			
QOL: Physical	≤10 years	59.51 ± 22.24		−2.339	0.019 *
	>10 years	62.26 ± 21.34			
QOL: Environmental	≤10 years	61.40 ± 20.44		−2.130	0.033 *
	>10 years	63.69 ± 19.41			
	Currently suffer from COVID-19 symptoms (n: 1200 no, 172 yes)		1370		
COVID-19 Contamination Anxiety	No	12.01 ± 8.91		−5.996	<0.001 ***
	Yes	16.42 ± 9.75			
COVID-19 Mental Health Impact: PTSD	No	9.00 ± 6.55		−5.006	<0.001 ***
	Yes	12.02 ± 7.52			
COVID-19 Mental Health Impact: Sleep Disturbances	No	5.09 ± 6.17		−6.414	<0.001 ***
	Yes	8.91 ± 7.47			
COVID-19 Mental Health Impact: Substance Use	No	4.07 ± 5.27		−5.358	<0.001 ***
	Yes	6.85 ± 6.53			
COVID-19 Stress Burden	No	17.09 ± 14.56		−6.586	<0.001 ***
	Yes	25.84 ± 16.53			
COVID-19: Conspiracy Beliefs	No	10.22 ± 5.49		−2.904	0.004 **
	Yes	11.54 ± 6.11			
Mental Fatigue	No	7.59 ± 8.27		−5.012	<0.001 ***
	Yes	11.45 ± 9.60			
Stigma of Social Discrimination	No	23.59 ± 7.31		−2.740	0.007 **
	Yes	25.42 ± 8.30			
QOL: Physical	No	61.78 ± 21.49		2.991	0.003 **
	Yes	56.48 ± 23.27			
QOL: Psychological	No	63.43 ± 20.05		2.932	0.003 **
	Yes	58.60 ± 21.24			
QOL: Social	No	66.13 ± 24.17		2.400	0.017 *
	Yes	60.95 ± 26.80			
QOL: Environmental	No	63.24 ± 19.53		2.195	0.029 *
	Yes	59.36 ± 22.00			
	Positive COVID-19 symptoms (n: 528 no, 852 yes)		1378		
COVID-19 Stress Burden	No	17.14 ± 14.82		−2.015	0.044 *
	Yes	18.82 ± 15.22			
Mental Fatigue	No	7.47 ± 8.54		−2.296	0.022 *
	Yes	8.56 ± 8.64			
	Under quarantine (n: 1314 no, 69 yes)		1381		
COVID-19 Contamination Anxiety	No	12.29 ± 9.01		−5.015	<0.001 ***
	Yes	17.90 ± 9.86			
COVID-19 Adherence Measures	No	8.35 ± 2.68		−2.094	0.036 *
	Yes	9.04 ± 2.54			
COVID-19 Mental Health Impact: PTSD	No	9.19 ± 6.27		−3.704	<0.001 ***
	Yes	12.78 ± 7.91			
COVID-19 Mental Health Impact: Sleep Disturbances	No	5.33 ± 6.32		−5.038	<0.001 ***
	Yes	9.99 ± 7.54			
COVID-19 Mental Health Impact: Substance Use	No	4.19 ± 5.31		−5.307	<0.001 ***
	Yes	8.90 ± 7.27			
COVID-19 Stress Burden	No	17.66 ± 14.72		−4.974	<0.001 ***
	Yes	28.80 ± 18.30			
COVID-19 Positive Coping: Daytime Structure	No	5.07 ± 2.56		−2.921	0.004 **
	Yes	6.64 ± 3.02			
Mental Fatigue	No	7.94 ± 8.53		−3.900	<0.001 ***
	Yes	12.07 ± 9.41			
Stigma of Social Discrimination	No	23.67 ± 7.32		−2.680	0.009 **
	Yes	26.74 ± 9.36			
QOL: Environmental	No	62.90 ± 19.78		2.026	0.043 *
	Yes	57.93 ± 21.79			
	Remote working/studying (n: 1207 no, 176 yes)		1381		
COVID-19 Contamination Anxiety	No	12.34 ± 9.06		−2.382	0.017 *
	Yes	14.10 ± 9.54			
COVID-19 Mental Health Impact: PTSD	No	9.12 ± 6.72		−3.617	<0.001 ***
	Yes	11.08 ± 6.64			
COVID-19 Mental Health Impact: Sleep Disturbances	No	5.39 ± 6.37		−2.480	<0.014 *
	Yes	6.77 ± 6.96			
COVID-19 Mental Health Impact: Substance Use	No	4.21 ± 5.37		−3.386	0.001 **
	Yes	5.90 ± 6.30			
COVID-19 Stress Burden	No	17.55 ± 14.77		−3.953	<0.001 ***
	Yes	22.77 ± 16.58			
COVID-19 Positive Coping: Daytime Structure	No	5.69 ± 2.59		−2.332	0.020 *
	Yes	6.18 ± 2.64			
COVID-19: Conspiracy Beliefs	No	10.23 ± 5.67		−2.648	0.008 **
	Yes	11.42 ± 5.53			
Stigma of Social Discrimination	No	23.65 ± 7.37		−2.337	0.025 *
	Yes	25.00 ± 7.98			

Note 1: COVID-19 = corona virus disease, PTSD = post-traumatic stress disorder, QoL = quality of life. Note 2: non-significant differences in outcome variables across the demographics were not reported in this table. * Statistically significant at (α ≤ 0.05), ** statistically significant at (α ≤ 0.01), *** statistically significant at (α ≤ 0.001).

**Table 5:** One-way ANOVA of the tested variables on participants’ sociodemographic variables, profession, city of residency, and city of employment.

Outcome Variable	Independent Variable	df	F-Value	*p*-Value
	Profession (n: 229 physician, 1045 nurse, 101 others)	2, 1380		
COVID-19 Adherence Measures			9.703	<0.001 ***
COVID-19 Mental Health Impact: PTSD			11.718	<0.001 ***
COVID-19 Stress Burden			8.004	<0.001 ***
COVID-19 Positive Coping: Inner Strength			7.044	0.001 ***
QOL: Physical			8.553	<0.001 ***
QOL: Psychological			11.871	<0.001 ***
QOL: Social			11.055	<0.001 ***
QOL: Environmental			21.742	<0.001 ***
	City of residency (n: 174 Dubai, 207 Abu Dhabi, 636 Sharjah, 109 Fujairah, 45 Umm Al Quwain, 57 Ras Al Khaimah, 105 Ajman, 50 Al-Ain)	7, 1375		
COVID-19 Mental Health Impact: PTSD			3.034	0.004 **
COVID-19 Stress Burden			3.356	0.001 ***
COVID-19 Positive Coping: Inner Strength			2.398	0.019 *
Stigma of Social Discrimination			3.036	0.004 **
QOL: Physical			2.616	0.011 *
QOL: Psychological			5.330	<0.001 ***
QOL: Social			4.183	<0.001 ***
QOL: Environmental			6.321	<0.001 ***
	City of employment (n: 176 Dubai, 211 Abu Dhabi, 722 Sharjah, 96 Fujairah, 45 Umm Al Quwain, 52 Ras Al Khaimah, 33 Ajman, 48 Al-Ain)	7, 1375		
COVID-19 Mental Health Impact: PTSD			2.266	0.027 *
COVID-19 Stress Burden			3.345	0.002 **
COVID-19 Positive Coping: Inner Strength			2.410	0.019 *
COVID-19: Conspiracy Beliefs			2.077	0.043 *
Stigma of Social Discrimination			3.100	0.003 **
QOL: Physical			2.732	0.008 **
QOL: Psychological			5.107	<0.001 ***
QOL: Social			4.082	<0.001 ***
QOL: Environmental			6.111	<0.001 ***

Note 1: COVID-19 = corona virus disease, PTSD = post-traumatic stress disorder, QoL = quality of life. Note 2: The table presents only the significant mean differences in outcome variables across the demographic groups. The non-significant differences were omitted for briefness. The table includes the F-values and *p*-values for each significant mean difference of each tested variable, indicating the statistical significance level. For each demographic variable, the mean scores of the outcome variables (mental health, quality of life, and stigma) are compared across different groups (profession, city of residency, and city of employment). Significant results highlight the specific groups where the differences were observed. * Statistically significant at (α ≤ 0.05), ** statistically significant at (α ≤ 0.01), *** statistically significant at (α ≤ 0.001).

**Table 6:** Correlation matrix of mental health, mental fatigue, stigma of the social discrimination, and quality of life (n = 1383).

	Age	CA	AM	PTSD	SD	SU	SB	DS	SC	IS	CB	MF	SSD	QOL-PH	QOL-PS	QOL-SO	QOL-EN
Age	1																
CA	−0.111 **	1															
AM	0.038	0.222 **	1														
PTSD	−0.128 **	0.544 **	0.277 **	1													
SD	−0.187 **	0.551 **	0.157 **	0.693 **	1												
SU	−0.188 **	0.543 **	0.170 **	0.686 **	0.837 **	1											
SB	−0.231 **	0.633 **	0.222 **	0.681 **	0.680 **	0.739 **	1										
DS	0.096 **	0.195 **	0.206 **	0.237 **	0.225 **	0.300 **	0.376 **	1									
SC	0.061*	0.160 **	0.221 **	0.267 **	0.183 **	0.243 **	0.296 **	0.662 **	1								
IS	0.145 **	0.074 **	0.151 **	0.081 **	0.017	0.052	0.107 **	0.460 **	0.516 **	1							
CB	−0.025	0.336 **	0.169 **	0.379 **	0.350 **	0.386 **	0.440 **	0.354 **	0.374 **	0.290 **	1						
MF	−0.213 **	0.434 **	0.032	0.381 **	0.545 **	0.491 **	0.479 **	0.063 *	0.021	−0.026	0.243 **	1					
SSD	−0.113 **	0.180 **	0.016	0.259 **	0.209 **	0.236 **	0.248 **	0.095 **	0.077 **	−0.64 *	0.186 **	0.166 **	1				
QOL-PH	0.212 **	−0.326 **	−0.027	−0.302 **	−0.422 **	−0.391 **	−0.380 **	0.069 *	0.102 **	0.168 **	−0.100 **	−0.460 **	−0.089 **	1			
QOL-PS	0.211 **	−0.297 **	−0.025	−0.311 **	−0.419 **	−0.407 **	−0.383 **	0.053 *	0.085 **	0.231 **	−0.084 **	−0.424 **	−0.129 **	0.849 **	1		
QOL-SO	0.184 **	−0.257 **	−0.038	−0.275 **	−0.355 **	−0.347 **	−0.340 **	0.024	0.040	0.167 **	−0.077 **	−0.351 **	−0.072 **	0.846 **	0.755 **	1	
QOL-EN	0.200 **	−0.275 **	−0.054 *	−0.308 **	−0.359 **	−0.364 **	−0.391 **	0.041	0.076 **	0.205 **	−0.052	−0.355 **	−0.113 **	0.863 **	0.850 **	0.847 **	1

CA: contamination anxiety, AM: adherence measures, PTSD: post-traumatic stress syndrome, SD: sleep disturbances, SU: substance use, SB: stress burden, DS: daytime structure, SC: social contact, IS: inner strength, CB: conspiracy belief, MF: mental fatigue, SSD: stigma of social discrimination, QOL: quality of life, QOL-PH: QOL Physical: QOL-PS: QOL Psychological, QOL-SO: QOL Social, QOL-EN: QOL Environmental. * Statistically significant at (α ≤ 0.05), ** statistically significant at (α ≤ 0.01).

## Data Availability

The data presented in this study are available on request from the corresponding author.
